# Bioinformatics-based analysis and experimental validation of PANoptosis-related biomarkers and immune infiltration in diabetic nephropathy

**DOI:** 10.3389/fendo.2025.1610882

**Published:** 2025-09-01

**Authors:** Su Zhang, Yun Zhang, Weitao Hu, Chunyan Huang, Yifang Zhang, Xiaoqing Chen

**Affiliations:** ^1^ The Second Clinical College of Fujian Medical University, Quanzhou, China; ^2^ Department of Rheumatology, The Second Affiliated Hospital of Fujian Medical University, Quanzhou, China; ^3^ Department of Nephrology, The Second Affiliated Hospital of Fujian Medical University, Quanzhou, China; ^4^ Department of Gastroenterology, The Second Affiliated Hospital of Fujian Medical University, Quanzhou, China; ^5^ Department of General Practice, The Second Affiliated Hospital of Fujian Medical University, Quanzhou, China

**Keywords:** diabetic nephropathy, PANoptosis, immune infiltration, biomarker, bioinformatics analysis

## Abstract

**Background:**

Diabetic nephropathy (DN) is a frequent and serious microvascular complication of diabetes. PANoptosis is a novel mode of cell death that encompasses apoptosis, necrosis and pyroptosis. However, effective PANoptosis-related biomarkers for DN are currently lacking. Therefore, this study aimed to elucidate the role of PANoptosis-related genes (PRGs) in the development of DN and their potential as diagnostic markers of DN, as well as their association with immune cell infiltration.

**Materials and methods:**

We retrieved the DN-related dataset GSE30122 from the GEO database. Then differentially expressed genes (DEGs) were identified and DEGs were analyzed for functional enrichment. In addition, we obtained key gene modules by WGCNA. Subsequently, we gained the intersecting genes of DEGs, key gene modules and PRGs. Four algorithms were further used to screen the key DE-PRGs in DN (DNDE-PRGs). We also investigated the biological functions of the key DNDE-PRGs by GSEA software. Furthermore, we analyzed the immune infiltration of DN tissues. The correlation of key genes with glomerular filtration rate (GFR) and blood urea nitrogen (BUN) was also examined. Finally, key genes were validated using clinical samples and db/db mice.

**Results:**

We identified two key DNDE-PRGs (*AKT3* and *FYN*). They showed good diagnostic value in the DN. And they were associated with immune cell infiltration. In addition, they have a correlation with GFR and BUN. Finally, they were validated in clinical samples and animal experiments.

**Conclusion:**

*AKT3* and *FYN* may be good PANoptosis-related biomarkers in DN. This provides new insights into the pathogenesis of DN.

## 1 Introduction

The prevalence of diabetes mellitus (DM) which is characterized by abnormally high blood glucose levels, is growing every year as the standard of living improves and global aging increases (1). There are approximately 537 million people with DM worldwide in 2021, representing 10.5% of the total global population (2). Diabetic nephropathy (DN) is one of the common microvascular complications of DM, as well as one of the leading causes of end-stage renal disease (3). Renal failure seriously affects the quality of life of patients and even threatens their lives and brings about psychological problems such as anxiety and depression. The development of DN is closely related to a persistent hyperglycemic state. Chronic hyperglycemia can lead to sclerosis of the vessel walls within the kidneys and an increased risk of cardiovascular diseases, which puts enormous economic pressure on individuals, families and society (4). Current treatment of DN relies heavily on the regulation of blood glucose, blood pressure, lipids, and the application of angiotensin-converting enzyme inhibitors, which have been effective in stopping the disease progression of DN (5). However, not all patients are well treated due to individual variability, difficulties in early diagnosis of DN and drug side effects (6). Currently, clinical diagnosis of DN still depends on the level of proteinuria, but this approach still has some false negatives (7, 8). Therefore, biomarkers for the early diagnosis of DN must be sought to facilitate early detection and intervention of DN.

PANoptosis was first proposed in 2019 by American scholars Malireddi et al. It involves all three modes of programmed cell death (PCD) simultaneously: pyroptosis, apoptosis, and necrosis (9). PANoptosis is activated by multiple signaling stimuli (e.g., DNA damage and oxidative stress), and the formation of PANoptosome initiates the entire process, leading to cell death (10). In recent years, many studies have reported the role of PANoptosis in many diseases, including infectious diseases, tumors, and cardiovascular diseases (10). In addition, the effect of PANoptosis in a variety of renal diseases is becoming clearer. High expression of PANoptosis is positively related to renal injury, and inhibition of the formation of PANoptosome helps to reduce renal injury (11). In renal tumors, PANoptosis has the potential to determine their prognosis (11). In hyperglycemic environments, PANoptosis integrates apoptosis, pyroptosis, and necroptosis through PANoptosome assembly, triggering synergistic inflammatory cascades, oxidative stress amplification, and tubulointerstitial fibrosis—key drivers of DN progression (12, 13). However, the role of PANoptosis-related genes (PRGs) in DN remains largely unknown. The infiltration of immune cells is an important factor in the development of DN. The accumulation of multiple myeloid cells in the kidney is closely associated with renal inflammation and injury (14).

In the present study, we performed bioinformatics analysis aiming to identify differentially expressed PRGs in DN (DNDE-PRGs) and screened key DNDE-PRGs using multiple algorithms. Further, we validated the expression of key DNDE-PRGs by clinical samples and *in vivo* experiments. In addition, we explored the relationship between key DNDE-PRGs and immune cell infiltration. In summary, key DNDE-PRGs may be useful for the early diagnosis and treatment of DN. The specific flow chart of the study is presented in **Supplementary Figure S1**.

## 2 Materials and methods

### 2.1 Acquisition of datasets and PRGs

The GEO (Gene Expression Omnibus, https://www.ncbi.nlm.nih.gov/geo/) database (15) was searched with the keywords “Diabetic nephropathy” or “Diabetic kidney disease” to obtain DN-related datasets. The selection criteria were as follows: 1. the species was Homo sapiens, 2. all samples were glomerular tissues, and 3. the samples contained both DN glomerular tissues and normal control glomerular tissues (NC). After excluding the tubular tissues, we finally chose three datasets, GSE30122, GSE30528, and GSE96804, as the data sources for analysis in this study. Their chip platforms were GPL571, GPL571 and GPL17586, respectively (**Table 1**). GSE30122 as the training set and the other two as the validation cohort. There were 930 PRGs obtained from previous reports (16) (**Supplementary Table S1**).

**Table 1:** Details of the datasets included in this study.

Dataset	Platform	Species	Tissue	Number of cases and controls	Type of cohortts
GSE30122	GPL571	Homo sapiens	Glomerular tissues	9 DN/26NC	Training
GSE30528	GPL571			9 DN/13NC	Validating
GSE96804	GPL17586			41DN/20 NC	Validating

### 2.2 Preprocessing of GSE30122 and identification of differentially expressed genes

We carried out residue completion, background correction and normalization of GSE30122 with the “limma” package (17). And principal component analysis (PCA) was performed on GSE30122 before and after normalization. Then we filtered out the differentially expressed genes in DN (DEGs). The criteria for screening were set as corrected *p* < 0.05 and ┃log2FoldChange┃ > 1.

### 2.3 Enrichment analysis of DEGs

GO analysis can characterize genes and their product functions. While KEGG analysis can link genes to numerous metabolic pathways, resulting in a more comprehensive understanding of biological system function. The enrichment analysis described above was realized through the Metascape website (https://metascape.org/) (18).

### 2.4 Identification of DN key gene modules

In order to identify the key gene modules of DN, we performed weighted gene co-expression network analysis using the “WGCNA” package (19). Based on the scale free topology model fit (R^2^ > 0.85), the optimum soft threshold (β) is firstly computed via the “pickThreshold” function. Then the gene module clustering tree was plotted and gene modules with block spacing less than 0.3 were merged. Finally, Pearson’s correlation coefficients (R) were calculated for every module with the clinical features of DN, thus identifying the key gene modules.

### 2.5 Identification of DNDE-PRGs

We took the intersection of DEGs, key gene modules and PRGs to obtain DNDE-PRGs. We then visualized the expression of DNDE-PRGs in the DN and NC groups using heatmap.

### 2.6 Identification of key DNDE-PRGs by multiple algorithms

The DNDE-PRGs were uploaded to the STRING database (https://cn.string-db.org/) (20), the minimum contribution score was set to 0.4, and the PPI network was plotted. Then visualize it with Cytoscape software (version 3.9.1) (21). The top 9 genes in terms of importance were acquired using both the maximal clique centrality (MCC) and maximum neighborhood component (MNC) algorithms of Cytoscape. The least absolute shrinkage and selection operator (LASSO) regression is a linear regression method for feature selection by adding an L1 regularization term to the loss function, which induces sparsification of the model coefficients, and is suitable for high-dimensional data analysis (22). The support vector machine-recursive feature elimination (SVM-RFE) is a feature selection method, based on the support vector machine (SVM) model, that progressively filters out the most important features by recursively removing the features that have the least impact on classification performance (23). The overlapping genes of the above four algorithms are the key DNDE-PRGs we need.

### 2.7 Diagnostic efficacy assessment and validation of key DNDE-PRGs

The diagnostic efficacy of the key DNDE-PRGs for DN was evaluated by plotting violin plots and receiver operating characteristic (ROC) curves from the training and validation cohorts. The ROC curves evaluate diagnostic efficacy by the area under the curve (AUC). The AUC of 0.7-0.8 is regarded as good; 0.8-0.9 is regarded as excellent; and more than 0.9 is regarded as outstanding. The two-sample t-test was applied to compare gene expression levels between the DN and NC groups.

### 2.8 Clinical samples validate expression of key DNDE-PRGs

#### 2.8.1 Samples collection

The renal puncture samples were acquired from 16 DN patients. The adjacent tissues of 28 renal carcinoma/renal cyst served as a control group (Control). We also collected gender, age, and relevant clinical and laboratory indicators for all participants (**Table 2**).

**Table 2:** Clinical traits of control and DN patients.

Clinical traits*	Control (n = 28)	DN (n = 16)
Sex, male/female	18/10	10/6
Age (year)	54.6 ± 12.5	52.6 ± 11.0
Duration (year)	NA	10.63 ± 7.29
Body weight (kg)	60.9 ± 11.2	61.2 ± 10.1
Height (cm)	161.7 ± 10.5	160.9 ± 9.8
BMI (kg/m^2^)	23.8 ± 3.56	22.9 ± 4.57
FBG (mmol/L)	5.4 ± 0.6	6.7 ± 2.2^##^
Hemoglobin (g/L)	132 ± 19	128 ± 18
Albumin (g/L)	46 ± 2	39 ± 3^#^
HbA1c (%)	NA	8.5 ± 3.0
HbA1c (mmol/mol)	NA	69 ± 9
CKD-EPI (ml/min/1.73m2)	93.1 ± 20.9	80.3 ± 27.5
Serum creatinine (μmol/L)	70 ± 22	100 ± 48
UAE (mg/day)	10.6 ± 7.0	1025 ± 543^###^
BUN (mmol/L)	5.4 ± 1.7	6.0 ± 1.9

*DN, diabetic nephropathy; BMI, body mass index; FBG, fasting blood glucose; CKD-EPI, chronic kidney disease epidemiology collaboration equations estimate glomerular filtration rate; UAE, urinary albumin excretion; BUN, blood urea nitrogen; NA, not available; ^#^ *P*<0.05 *vs*. control group; ^##^ *P*<0.01 *vs*. control group; ^###^ *P*<0.001 *vs*. control group.

#### 2.8.2 Immunofluorescence (IF) staining

Tissue samples were first fixed and sectioned, then the fixative was removed and the non-specific binding sites were closed by a blocking solution. Next, a primary antibody (AKT3: ab152157, abcam; FYN: ab184276, abcam) was added to bind to the target antigen, incubated and washed to remove unbound primary antibody. Subsequently, fluorescently labeled secondary antibody (IgG, SA00001-15, proteintech) were added and incubation was continued and washed to remove unbound secondary antibodies. Finally, staining was performed and fluorescence signals were observed using a fluorescence microscope to achieve detection and localization of the target antigen.

#### 2.8.3 Real-time quantitative reverse transcription polymerase chain reaction (RT-qPCR)

First, total RNA was extracted from tissue samples using an RNA extraction kit. Then, RNA was reverse transcribed to cDNA with reverse transcriptase. Next, specific primers and fluorescent probes were used for real-time quantitative PCR reactions. During PCR amplification, fluorescent signals were monitored in real time to reflect the amount of target gene amplification by changes in fluorescence intensity. The accumulation of fluorescent signal during each cycle was proportional to the initial expression of the target gene. Finally, by analyzing the cycling threshold (Ct value) of the fluorescence signal, the relative expression level of the target gene in the tissue can be quantified. The housekeeping gene GAPDH was applied to normalize target genes’ CT value. The primer sequences for this section are presented in **Table 3**.

### 2.9 Correlation analysis of key DNDE-PRGs with clinical traits of DN

The Nephroseq v5 database (http://v5.nephroseq.org/) (24) contains clinical and gene expression data for a wide range of renal diseases. We analyzed Pearson correlations between key DNDE-PRGs and clinical traits of DN.

### 2.10 Immune infiltration analysis

Cibersort (https://cibersortx.stanford.edu/) (25) is a method for analyzing immune cell infiltration using an inverse convolution algorithm. It uses gene expression signatures of specific immune cell types to infer the relative abundance of different immune cells in a sample. It compares the overall gene expression data of the sample with known gene expression profiles of immune cells to quantitatively infer the proportion of each type of immune cell (e.g., T cells, B cells, macrophages, etc.) in the sample. In addition, we analyzed the Pearson correlation of key DNDE-PRGs with immune-infiltrating cells, with ┃R┃ ≥ 0.6 and *p* < 0.05 representing statistical significance.

### 2.11 Gene set enrichment analysis (GSEA) of key DNDE-PRGs

SEA is a method for evaluating significant enrichment in gene expression data by gene sets. The principle is to rank all genes in terms of their expression differences, and then evaluate the enrichment of a predefined set of genes in the ranked list to determine whether the set is significantly different between the experimental group and the control group. The benefit of GSEA is the ability to identify signals that may not be significant at the level of individual genes but are biologically important at the level of sets of genes, thus helping to reveal underlying biological processes and mechanisms, and is particularly suited to the analysis of complex biological systems (26).

### 2.12 Construction of an interaction network of key DNDE-PRGs with PANoptosis marker genes

We subsequently constructed an interaction network between key DNDE-PRGs and some previously reported PANoptosis marker proteins in the STRING database (https://cn.string-db.org/) (20). This manipulation was utilized to enhance the link between key DNDE-PRGs and PANoptosis. PANoptosis marker genes sourced from references (27–34).

### 2.13 Animal experiments validate key DNDE-PRGs

#### 2.13.1 Animal selection & grouping

Six-week-aged db/m and db/db mice were purchased from Shanghai SLAC Co. The db/db mouse is the commonly used model of spontaneous DN, while the db/m mouse is commonly used as its control. Divide them into db/m group (n=6) and db/db group (n=6). Mice were housed in individual metabolic cages for 24h urine collection every two weeks. Urine was stored at -80°C for further analysis. Mice were sacrificed at 12th week. Kidney and blood samples were collected under anesthesia, and stored at -80°C for further analysis.

#### 2.13.2 Measurement of relevant biochemical indicators

The Glucose Assay Kit (S0201M, Beyotime) was utilized to measure fasting blood glucose (FBG) concentration in each mouse. Urinary albumin levels were quantified using a mouse albumin-specific ELISA kit (ab108792, Abcam). Urinary albumin excretion (UAE) was calculated as urinary albuminuria (mg/mL) ✖ urine volume (mL)/24h. Urea Assay Kit (C013-2-1, Nanjing Jiancheng Bioengineering Institut) was designed to detect blood urea nitrogen (BUN) levels.

#### 2.13.3 Periodic acid-Schiff staining

Tissue sections were first deparaffinized and hydrated, followed by oxidation of the carbohydrate in the sections with periodic acid. Subsequently, the sections were stained with Schiff’s reagent, with the reaction of the carbohydrates with Schiff’s reagent producing a purplish or dark red complex. This is followed by contrast staining using hematoxylin, which gives a blue color to the nuclei, and finally by gradient alcohol dehydration, xylene clearing and sealing the sections with neutral resin.

#### 2.13.4 Masson staining

The tissue sections were first deparaffinized and hydrated, then stained with Regaud’s hematoxylin stain. Next, they are stained with acidic magenta and then treated with phosphomolybdic acid solution to remove excess magenta. Finally, the collagen fibers were stained blue with gentian violet stain. After dehydration, clearing and sealing, Masson staining was completed.

***IF staining and RT-qPCR*** steps were as in 2.8. The primers sequences used in this section are presented in **Table 3**.

**Table 3:** The primers sequences used in this study.

Gene names	Primers sequences (5’→3’)
GAPDH-F (human)	GCACCGTCAAGGCTGAGAAC
GAPDH-R (human)	TGGTGAAGACGCCAGTGGA
AKT3-F (human)	TGTGGATTTACCTTATCCCCTCA
AKT3-R (human)	GTTTGGCTTTGGTCGTTCTGT
FYN-F (human)	ATGGGCTGTGTGCAATGTAAG
FYN-R (human)	GAAGCTGGGGTAGTGCTGAG
GAPDH-F (mouse)	AGGTCGGTGTGAACGGATTTG
GAPDH-R (mouse)	TGTAGACCATGTAGTTGAGGTCA
AKT3-F (mouse)	TGGGTTCAGAAGAGGGGAGAA
AKT3-R (mouse)	AGGGGATAAGGTAAGTCCACATC
FYN-F (mouse)	ACCTCCATCCCGAACTACAAC
FYN-R (mouse)	CGCCACAAACAGTGTCACTC

### 2.14 Statistical analysis

All the above analyses were performed in R software (version 4.4.3). Differences between the two samples were processed using GraphPad Prism 10 software and compared using the student’s t test. The data with *p* < 0.05 were deemed significant.

## 3 Results

### 3.1 Identification of DEGs

**Figure 1:**
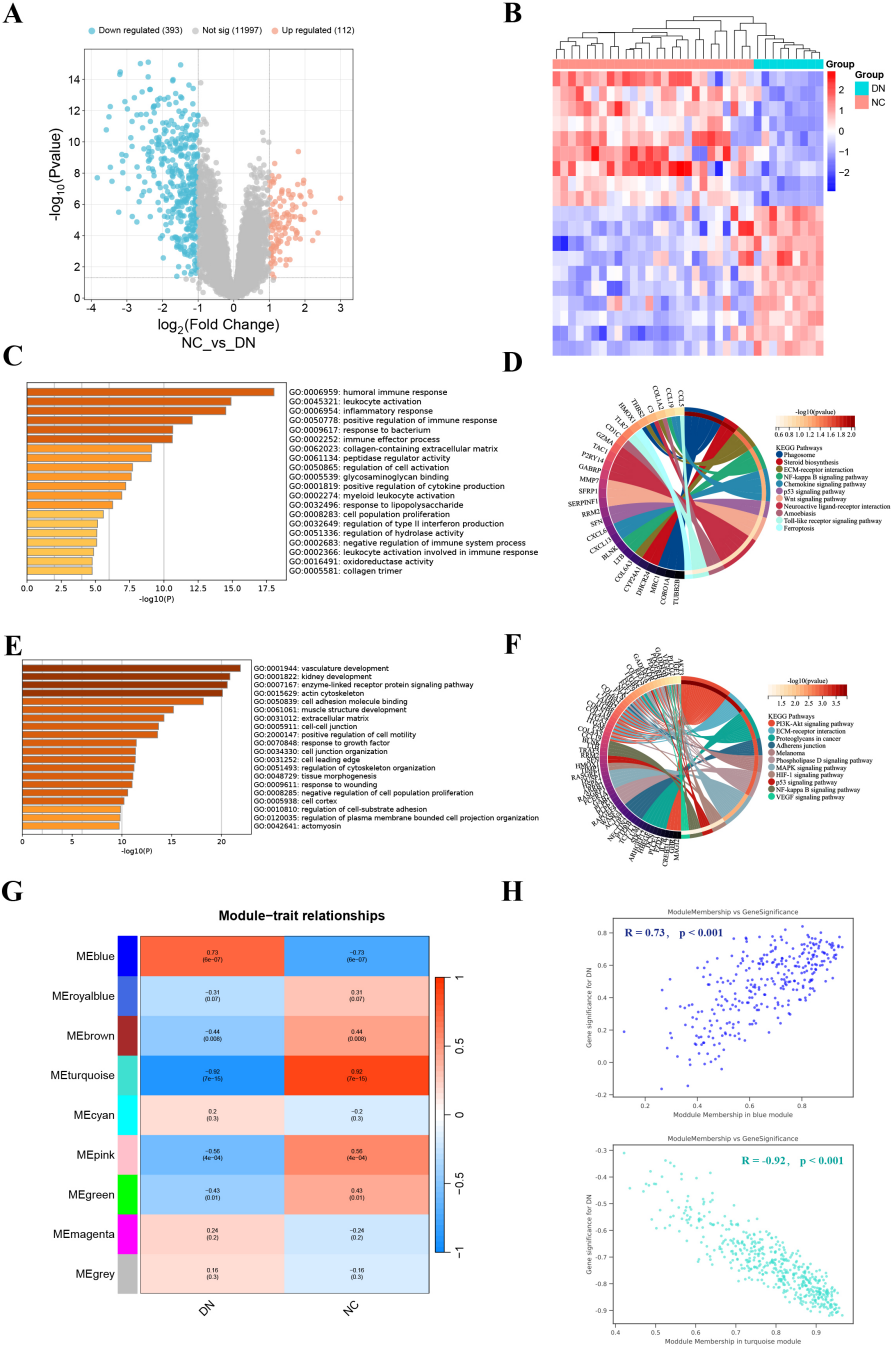
Identification and enrichment analysis of DEGs, and WGCNA analysis. **(A)** Volcano plot displayed 505 DEGs in DN, including 112 up-regulated genes and 393 down-regulated genes. **(B)** The heatmap showed the 10 up-regulated genes and 10 down-regulated genes with the most significant differences. **(C, D)** GO (left) and KEGG (right) enrichment analysis of up-regulated DEGs. **(E, F)** GO (left) and KEGG (right) enrichment analysis of down-regulated DEGs. **(G)** Correlation heatmap of each module with clinical phenotypes of DN. **(H)** Scatterplot of correlation between key gene module membership and gene significance.

The PCA results showed that the normalized GSE30122 intra-group gap was significantly reduced, which facilitated the subsequent analysis (**Supplementary Figure S2**). A total of 505 DEGs were screened according to the previously described screening criteria, of which 112 were up-regulated genes and 393 were down-regulated genes. The DEGs were visualized utilizing a volcano plot. (**Figure 1A**). The heatmap showed the 10 up-regulated genes and 10 down-regulated genes with the most significant differences (**Figure 1B**).

### 3.2 Functional enrichment analysis of DEGs

To probe the DEGs-related biological functions, GO and KEGG enrichment analyses were conducted for up-regulated DEGs and down-regulated DEGs. GO analysis indicated that up-regulated DEGs were participated in multiple immune and inflammatory responses, including humoral immune response, leukocyte activation, and inflammatory response (**Figure 1C**). While KEGG analysis revealed that up-regulated DEGs were most significantly enriched in phagosome (**Figure 1D**). GO terms for down-regulated DEGs include vascular development, kidney development, and enzyme-linked receptor protein signaling pathways (**Figure 1E**). In addition, they also engage in the PI3K-Akt signaling pathway (**Figure 1F**). In general, DEGs have abundant biological functions.

### 3.3 Acquisition of key gene modules

We found that β = 24 when R^2^ = 0.85 (**Supplementary Figure S3A**). A total of 9 modules were obtained after merging the gene modules (**Supplementary Figure S3B**). The blue module showed the strongest positive correlation with the DN clinical phenotype (R = 0.73); while the turquoise module exhibited the strongest negative correlation with the DN clinical phenotype (R = -0.92) (**Figures 1G, H**). Therefore, the blue and turquoise modules were regarded as key gene modules.

### 3.4 Identification of key DNDE-PRGs

**Figure 2:**
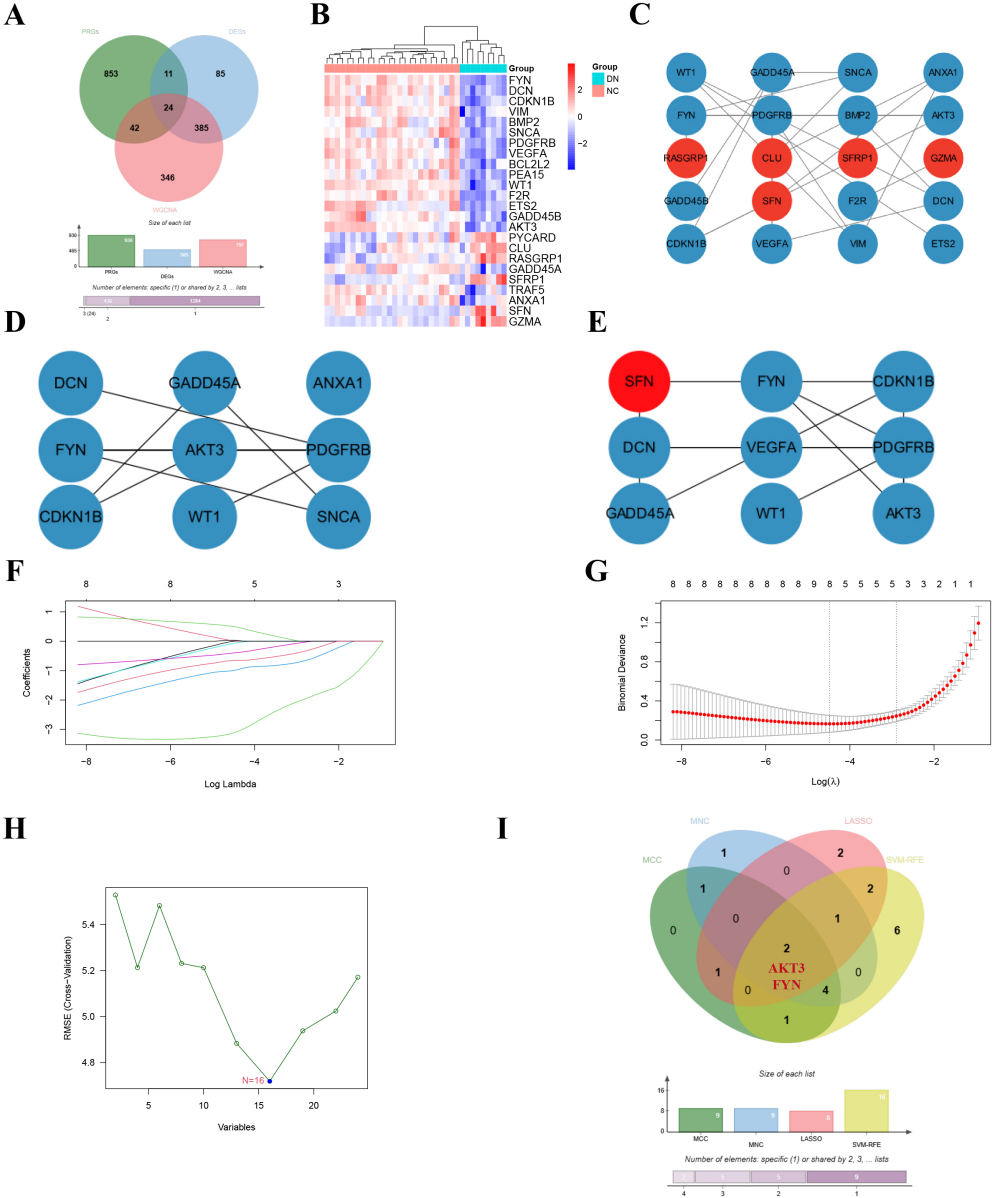
Identification of key DNDE-PRGs. **(A)** Venn diagram showed overlapping genes in DEGs, key gene modules and PRGs. **(B)** Heatmap revealed the distribution of the 24 DNDE-PRGs in the DN and NC groups. **(C)** Protein-protein interaction network of DNDE-PRGs, with red representing up-regulated genes and cyan representing down-regulated genes. **(D, E)** The top 9 genes in terms of importance obtained by the MCC algorithm (left) and the MNC algorithm (right). **(F, G)** LASSO regression analysis. **(H)** SVM-RFE machine learning algorithm. **(I)** Key DNDE-PRGs (AKT3 and FYN) derived from the four algorithms.

The 24 overlapping genes of DEGs, key gene modules and PRGs are the DNDE-PRGs (**Figure 2A**). These include 6 up-regulated genes and 18 down-regulated genes (**Figure 2B**). **Figure 2C** displayed their PPI networks, with red representing up-regulated genes and cyan representing down-regulated genes. **Figure 2D** and **Figure 2E** illustrate the top 9 genes in terms of importance obtained by the MCC algorithm and the MNC algorithm. To improve the accuracy of screening key DNDE-PRGs, we further applied LASSO regression (**Figures 2F, G**) and SVM-RFE (**Figure 2H**) machine learning algorithms. After combining the four algorithms, we get two key DNDE-PRGs, namely AKT3 and FYN (**Figure 2I**). The DNDE-PRGs identified by the four algorithms are illustrated in **Table 4**.

**Table 4:** Key DNDE-PRGs obtained by the four algorithms.

Algorithm names	Gene symbol	Overlapping genes
MCC	DCN, WT1, SNCA, GADD45A, FYN, AKT3, CDKN1B, ANXA1, PDGFRB	AKT3 FYN
MNC	SFN, PDGFRB, WT1, AKT3, FYN, DCN, GADD45A, CDKN1B, VEGFA	
LASSO	GZMA, F2R, BMP2, AKT3, FYN, ANXA1, RASGRP1	
SVM-RFE	FYN, DCN, CDKN1B, VIM, BMP2, SNCA, PDGFRB, VEGFA, BCL2L2, PEA15, WT1, F2R, ETS2, GADD45B, AKT3, PYCARD,	

### 3.5 ROC analysis and validation of key DNDE-PRGs

**Figure 3:**
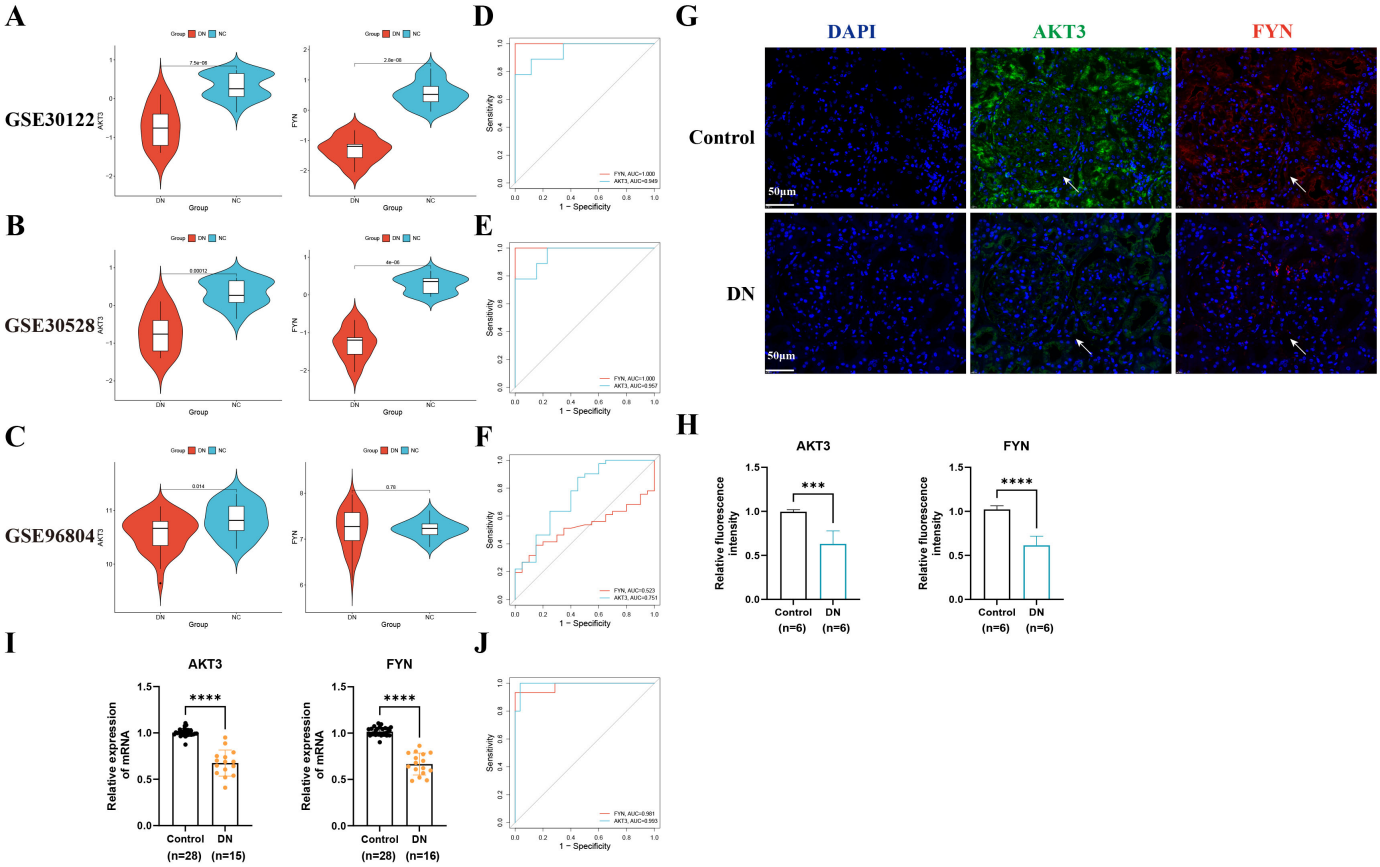
External datasets and clinical sample validation for key DNDE-PRGs. **(A–C)** Violin plots illustrated the expression of key DNDE-PRGs in the training and validation sets. **(D–F)** ROC analysis of key DNDE-PRGs in training and validation sets. **(G–H)** IF staining results (up) and relative fluorescence intensity (down) of key genes in clinical kidney tissue samples. **(I)** The relative mRNA expression levels of key DNDE-PRGs in clinical renal tissue samples. **(J)** The ROC curve was plotted based on RT-qPCR results. *** represents *p* < 0.001; **** represents *p* < 0.0001. Scale: 50 μm.

In order to test the accuracy of the above algorithms and the diagnostic efficacy of key DNDE-PRGs for DN, we carried out ROC and key gene expression analyses. We found that the key DNDE-PRGs were down-regulated in both the validation and training sets (**Figures 3A-C**). In addition, ROC analysis indicated that the key DNDE-PRGs had a good diagnostic value for DN, as demonstrated by the AUC > 0.5 (**Figures 3D-F**). Both IF staining and RT-qPCR results of kidney samples indicated decreased expression of AKT3 and FYN in DN (**Figures 3G-J**). This strongly supports the accuracy of our bioinformatics analysis results. These results suggest that AKT3 and FYN may be potentially good biomarkers for DN.

### 3.6 Correlation analysis between key DNDE-PRGs and clinical traits

**Figure 4:**
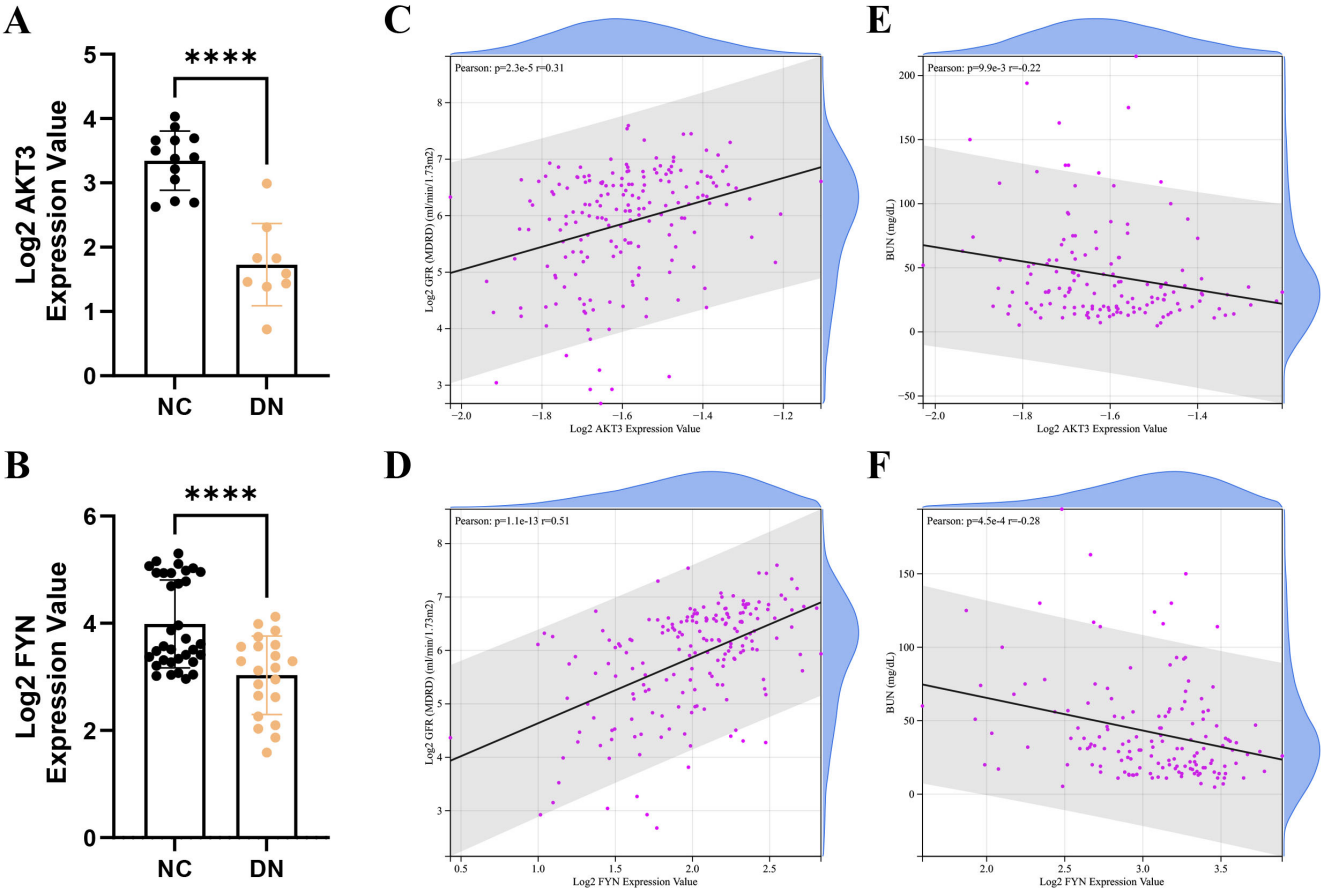
Correlation analysis of key DNDE-PRGs with clinical traits of DN. **(A, B)** Expression levels of key DNDE-PRGs in the DN and NC groups in the Nephroseq v5 database. **(C, D)** Pearson correlation of key DNDE-PRGs with glomerular filtration rate. **(E, F)** Pearson correlation of key DNDE-PRGs with blood urea nitrogen.

In the Nephroseq v5 database, we detected that the expression of AKT3 and FYN in DN group was significantly lower than that in NC group (**Figures 4A, B**). Interestingly, key DNDE-PRGs were positively correlated with glomerular filtration rate (GFR) (AKT3, r = 0.31; FYN, r = 0.51); and negatively correlated with blood urea nitrogen (BUN) (AKT3, r = -0.22; FYN, r = -0.28) (**Figures 4C-F**). This may suggest that decreased expression of key DNDE-PRGs favors DN progression.

### 3.7 Analysis of immune cell infiltration

**Figure 5:**
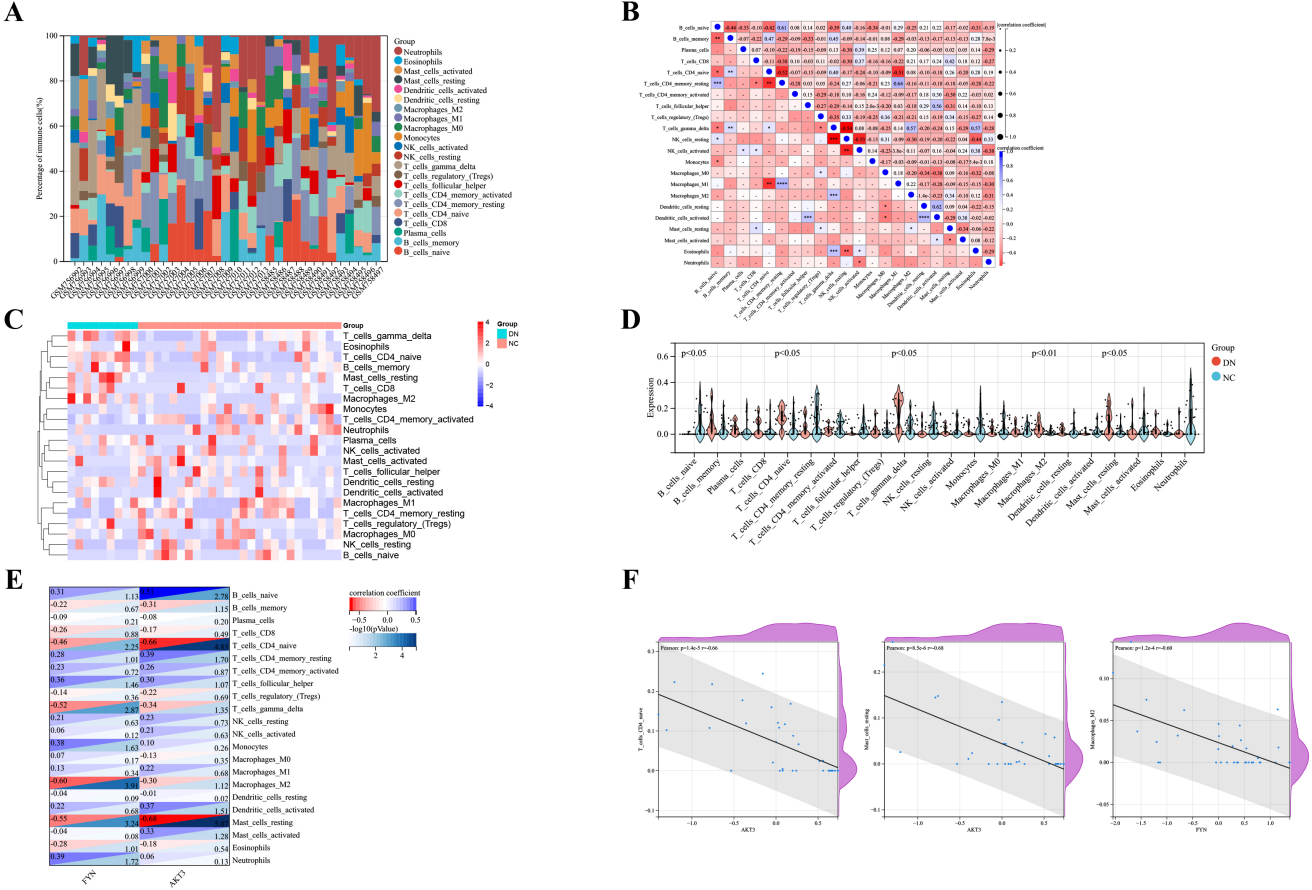
Analysis of immune infiltration in DN renal tissue. **(A)** Relative abundance of 22 immune cells in each sample of GSE30122. **(B)** Correlation analysis of immune cells. **(C)** Heatmap showed the relative expression levels of 22 immune cells in the DN and NC groups. **(D)** Violin plots displayed the difference in expression of 22 immune cells between the DN and NC groups. p<0.05 represents statistically significant. **(E)** Correlation analysis of key DNDE-PRGs with immune cell infiltration. **(F)** Scatterplots of correlation between key DNDE-PRGs and immune cell infiltration.

The Cibersort algorithm calculated the relative abundance of 22 immune cells in each sample (**Figures 5A, C**). Positive correlations were observed between resting CD4^+^ T memory cells and M1 macrophages (r = 0.64), resting and activated dendritic cells (r = 0.62), as well as naive B cells and resting CD4^+^ T memory cells (r = 0.61) (**Figure 5B**). Notably, five immune cells were significantly different in the DN and NC groups (*p* < 0.05). Expanding on this is that naive CD4^+^ T cells, γδT cells, M2 macrophages, and resting mast cells were more abundantly expressed in DN. In contrast, naive B cells were less expressed in DN (**Figure 5D**). Pearson correlation was further applied to detect the correlation between key DNDE-PRGs and immune cells. The AKT3 was negatively correlated with naive CD4^+^ T cells (r = -0.66) as well as resting mast cells (r = -0.68). And FYN was negatively correlated with M2 macrophages (r = -0.60) (**Figures 5E, F**).

### 3.8 Signaling pathways of key DNDE-PRGs

**Figure 6:**
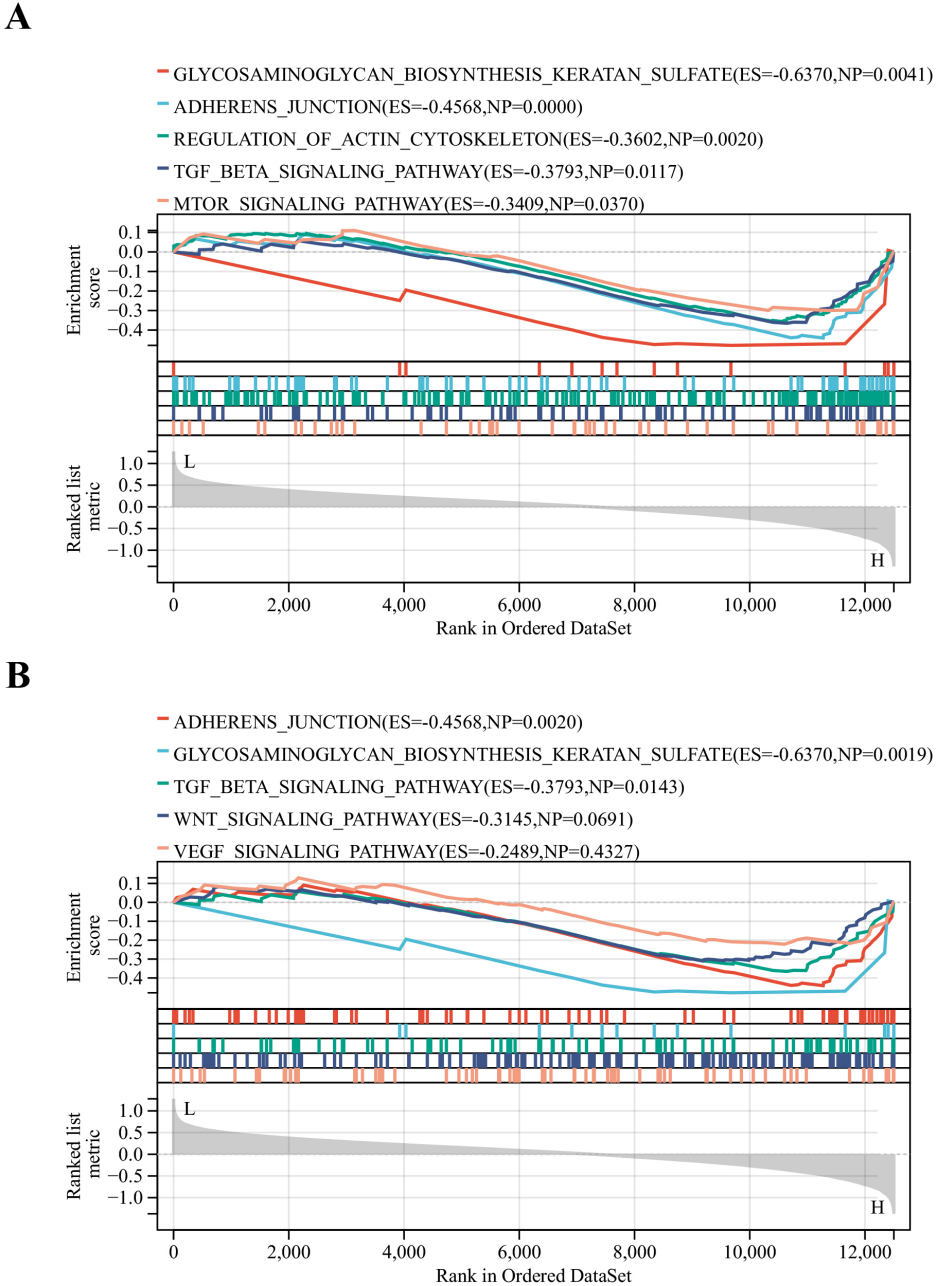
Identification of relevant pathways for key DNDE-PRGs. **(A)** GSEA enrichment analysis of AKT3. **(B)** GSEA enrichment analysis of FYN.

The DN patients were categorized into high and low expression groups based on the median expression values of key genes. Then GSEA analysis was performed. To our surprise, glycosaminoglycan biosynthesis keratan sulfate, adherens junction and TGF-β signaling pathways were common signaling pathways enriched in the AKT3 and FYN high-expression groups (**Figures 6A, B**). All of these pathways are inextricably linked to the development of diabetes and its associated complications (35–37).

### 3.9 Association of key DNDE-PRGs with PANoptosis

Our constructed interaction network displayed that both AKT3 and FYN were connected to CASP3, which implied that the key DNDE-PRGs might be involved in PANoptosis by regulating the expression of CASP3 (**Supplementary Figure S4**). At the same time, this also provides a direction for our follow-up study.

### 3.10 Verification of the expression of key DNDE-PRGs by animal experiments

**Figure 7:**
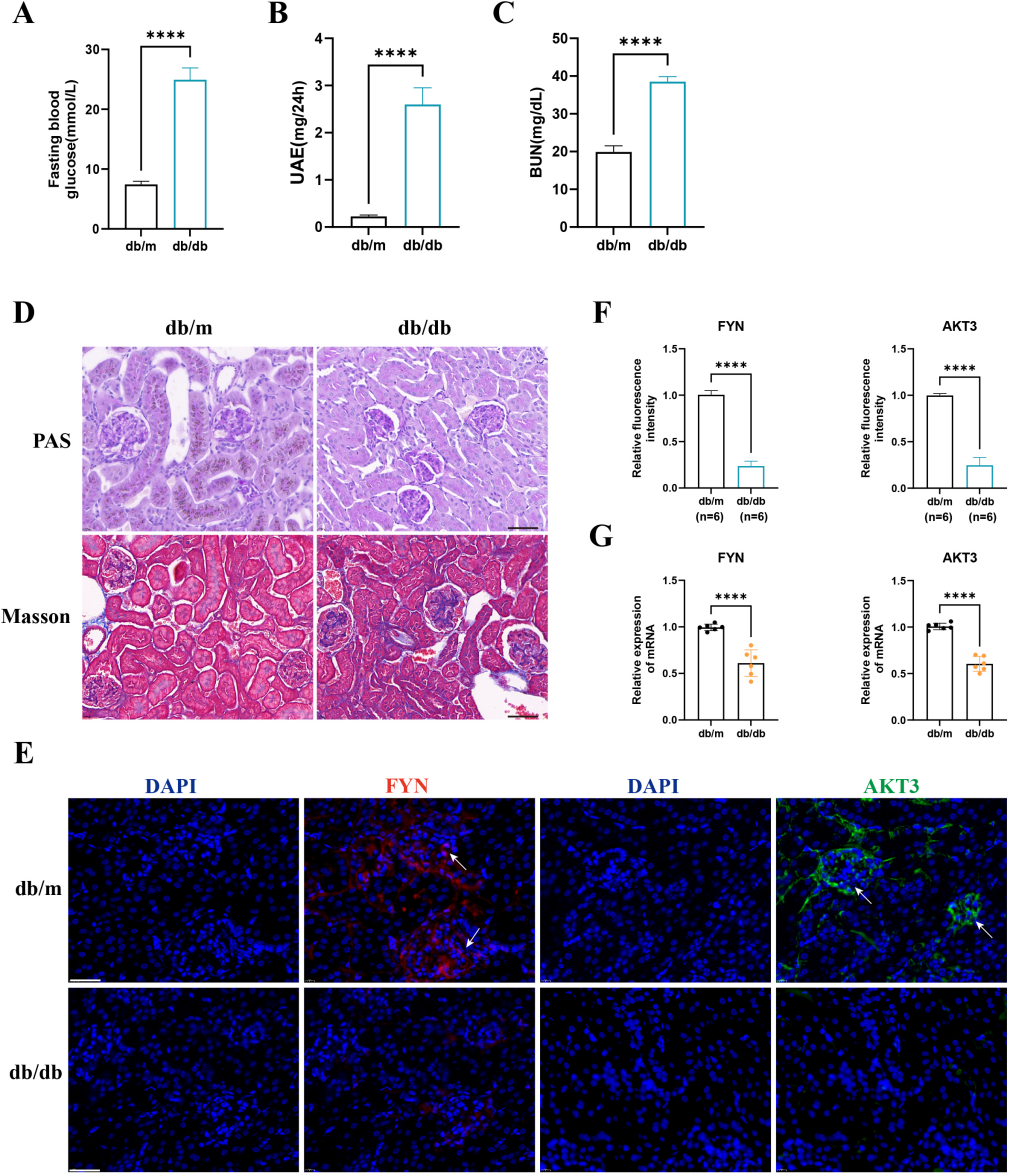
The expression of key DNDE-PRGs was verified by *in vivo* experiments. **(A–C)** Fasting blood glucose, urinary albumin excretion (UAE) and blood urea nitrogen (BUN) levels in db/m and db/db group mice. **(D)** PAS staining and Masson staining in db/m and db/db group mice. **(E, F)** IF staining and relative fluorescence intensity of key DNDE-PRGs in db/db group mice. **(G)** The relative mRNA expression levels of key DNDE-PRGs. **** represents *p* < 0.0001. Scale: 50 μm.

We observed that FBG, UAE, and BUN levels were significantly higher in the db/db group than in the db/m group (**Figures 7A-C**). PAS and Masson staining suggested glomerular atrophy, obvious inflammatory cell infiltration and fibrous tissue proliferation (**Figure 7D**). These strongly support the successful construction of the DN mouse model. IF staining suggested that AKT3 and FYN were predominantly expressed in glomeruli in the db/m group and were significantly higher than in the db/db group (**Figures 7E, F**). RT-qPCR results were consistent with IF staining (**Figure 7G**).

## 4 Discussion

DM is a globally prevalent metabolic disease that is usually associated with multiple complications. DN, as one of its typical microvascular complications, is an important part of renal failure. It is vital to explore the pathogenesis of DN and develop new treatments. There is growing evidence of the importance of multiple cell death patterns in the pathogenesis of DN (38–40). Whereas PANoptosis is an emerging PCD, its role in DN is still unknown.

PANoptosis is a novel PCD that has now been demonstrated to be engaged in the onset and progression of neurological disorders, inflammatory diseases and other diseases (41, 42). PANoptosis includes apoptosis, necrosis, and pyroptosis (9). Apoptosis is hyperactivated in the context of DN. Excessive apoptosis and pyroptosis of podocytes leading to low numbers has been established as an important mechanism in the development of DN (43). Inhibition of podocyte apoptosis and pyroptosis significantly ameliorates renal injury and reduces urinary albumin levels in a mouse model of DN (44, 45). In addition, hyperglycemia may trigger tubular necrosis to promote the development of DN (46). In the context of diabetes, cellular oxidative stress, inflammatory responses, and abnormal signaling pathway activation may disrupt the normal regulation of PANoptosis (47). The aberrant activation of PANoptosis in turn further promotes the persistence of inflammation, thus creating a vicious cycle and exacerbating the development of diabetes and its complications (13). Given the above evidence, we conjecture that PANoptosis is also closely related to the development of DN.

In this study, we identified DEGs in DN using GSE30122 and analyzed them for functional enrichment. WGCNA produced two key gene modules for DN. The overlap of DEGs, key gene modules and PRGs then generated 24 DE-PRGs. Subsequently, we identified two key DNDE-PRGs (AKT3 and FYN) by MCC algorithm, MNC algorithm, LASSO regression and SVM-RFE machine learning methods. GSEA enrichment analysis revealed that the common enrichment pathways for AKT3 and FYN include glycosaminoglycan biosynthesis keratan sulfate, adherens junction and TGF-β signaling pathways. Immune infiltration analysis showed that DN has a dysregulated immune microenvironment and that key DNDE-PRGs correlate with immune cell infiltration. ROC analysis suggested that key genes are of high value for the diagnosis of DN. Moreover, the key genes are correlated with the clinical traits of DN. Finally, the expression of key DNDE-PEGs was validated by clinical samples and animal experiments. To our knowledge, this is the first study on screening and validating key PRGs in DN.

*AKT3* (AKT Serine/Threonine Kinase 3) is a member of the AKT family. It is widely involved in cell growth, proliferation, survival and metabolism, as well as being an important mediator in the regulation of inflammatory and immune responses (48, 49). The AKT signaling pathway and its associated molecules have been considered to exert a critical function in the progression of DN (50–52). For example, AKT3 regulated by the circ_0037128/miR-17-3p axis promotes glomerular cell proliferation, fibrosis, inflammation, and oxidative stress, which in turn favors DN (53). Knockdown of AKT3 effectively attenuated high glucose-induced mesangial cell injury and blood glucose and UAE levels in DN mice (54). Notably, as a core component of the PI3K-AKT pathway, AKT3 interacts with key regulators including stimulator of interferon genes (STING) and phosphatase and tensin homolog (PTEN). STING-mediated inflammation has been implicated in renal tubular injury and fibrosis, while PTEN deficiency exacerbates DN progression through AKT hyperactivation (55, 56). These interactions may constitute additional molecular mechanisms through which AKT3 dysregulation contributes to DN pathogenesis. In addition, Zhang et al. found that Esculentoside H could alleviate PANoptosis and protect the blood-brain barrier through activation of the TLE1/PI3K/AKT signaling pathway in the context of cerebral ischemia/reperfusion (57). Meanwhile, Yang et al. observed that Dachaihu decoction inhibited PANoptosis through inhibiting the PI3K/AKT/NF-κB pathway, thereby attenuating sepsis-induced acute lung injury (58). However, direct evidence of the relationship between AKT3 and PANoptosis in the context of DN is still lacking.

*FYN* is a member of src family tyrosine kinase. The role of FYN in tumorigenesis has been extensively studied and has been demonstrated to promote tumor cell growth and migration (59, 60). DN mice with deficient FYN expression display reduced levels of renal oxidative stress and reduced matrix deposition (61). Nuclear receptor coactivator 3 (NCOA3) deficiency has been reported to drive podocyte damage in DN mice. While inhibition of FYN rescued this driving effect (62). FYN can activate NLRP3 inflammatory vesicles and amplify inflammation in Parkinson’s disease (63). In addition, the low expression of FYN favors the occurrence of autophagy in renal cells, which plays a protective role against DN (64, 65). However, there is a lack of reports on FYN and PANoptosis in the course of DN. Interestingly, while TEA domain transcription factor 1(TEAD1, a transcriptional regulator in the Hippo pathway) was not identified in our DNDE-PRGs screening, recent work demonstrates its critical role in driving necroptosis and inflammation in kidney injury via RIPK3-dependent signaling (66). Though mechanistically distinct from PANoptosis, TEAD1-mediated necroptosis may synergize with pyroptosis/apoptosis pathways in DN progression. Future studies should explore crosstalk between TEAD1 and PANoptosis regulators (e.g., ZBP1, NLRP3) in DN.

The pathogenesis of DN is intricately related to immune cell infiltration, with macrophage polarization playing a critical role in driving renal inflammation and fibrosis (67). Our data and recent studies indicate that: M1 macrophages predominate in the early stages of DN and exacerbate podocyte injury through the production of pro-inflammatory cytokines (TNF-α, IL-1β) (68, 69). M2 macrophages are commonly attributed with anti-inflammatory effects (70). M2 macrophage polarization effectively attenuates DN kidney injury (70, 71). This action is linked to a reduction in the secretion of several pro-inflammatory cytokines and pro-fibrotic proteins (72, 73). Macrophage plasticity allows for phenotypic switching according to the renal microenvironment. The hyperglycemic environment may maintain M1 polarization through overproduction of reactive oxygen species (74). As DN progresses it may drive M2 phenotypic switching, which may be a compensatory mechanism for the tissue (67). Therapeutically, promotion of M1 to M2 transition attenuates the progression of DN (67). The enrichment of M2 macrophages observed in our immune infiltration analysis of DN tissues is consistent with this injurious transition. We also observed that including naive CD4^+^ T cells and resting mast cells were more highly expressed in DN tissues. CD4^+^ T cell-mediated islet destruction is an important basis for the development of autoimmune diabetes (75). In addition, multiple CD4^+^ T cell subsets have a critical function in the progression of DN (76). And naive CD4^+^ T cells activate and differentiate into different effector T cells upon appropriate stimulation (77). Previous research has revealed that mast cells were significantly elevated in number and activity in DN mice and may be an essential driver of renal fibrosis (78). And mast cells were also thought to be closely related to kidney inflammation (79). Immune infiltration analysis suggests that the low expression of *AKT3* and *FYN* favors the infiltration of the above immune cells in the kidney thereby exacerbating renal injury in DN. Nevertheless, more studies are needed to investigate the role of these immune cells in DN.

It is worth noting that this study also has some limitations. Concretely, it is that the sample size included in the study was too small and included only a clinical sample from one center. Secondly, this study was a secondary mining in a public dataset, which may be biased. Thirdly, while our CIBERSORT-based analysis revealed significant correlations between AKT3/FYN expression and specific immune cell subsets (e.g., M2 macrophages, naïve CD4^+^ T cells), this proportional approach provides only a preliminary view of immune dysregulation in DN. We acknowledge that deeper characterization of the immune microenvironment is needed to fully elucidate how PANoptosis-related biomarkers shape immunological landscapes. Notably, while our study identified AKT3 and FYN as downregulated PANoptosis-associated genes in DN, we did not experimentally validate their mechanistic roles in coordinating apoptosis, pyroptosis, and necroptosis. Future studies should interrogate whether AKT3/FYN deletion or overexpression directly modulates PANoptosome assembly in diabetic renal cells.

## 5 Conclusion

In brief, this study integrated bioinformatics and machine learning to systematically analyze the biological meaning of PANoptosis-related genes in DN and its connection with immune cells. AKT3 and FYN were characterized as DN PANoptosis-related biomarkers. This provides a deeper insight into the pathogenesis of DN.

## Data Availability

The original contributions presented in the study are included in the article/Supplementary Material. Further inquiries can be directed to the corresponding author.

## Supplementary material

Supplementary Figure 1 Specific flow chart for this study.

Supplementary Figure 2 Three-dimensional stereogram of principal component analysis before and after normalization of GSE30122.

Supplementary Figure 3 Filtering for optimal soft thresholds **(A)** and modular clustering trees **(B)**.

Supplementary Figure 4 The protein interaction network of key DNDE-PRGs with PANoptosis marker genes. Red color represents key DNDE-PRGs. Green and purple color represent PANoptosis marker genes.
