# Supplementary figures and images for: Bioinformatics-based analysis and experimental validation of PANoptosis-related biomarkers and immune infiltration in diabetic nephropathy
Source: Front Endocrinol (Lausanne). 2025 Sep 1;16:1610882. doi: 10.3389/fendo.2025.1610882 (PMC12434962; doi:10.3389/fendo.2025.1610882)

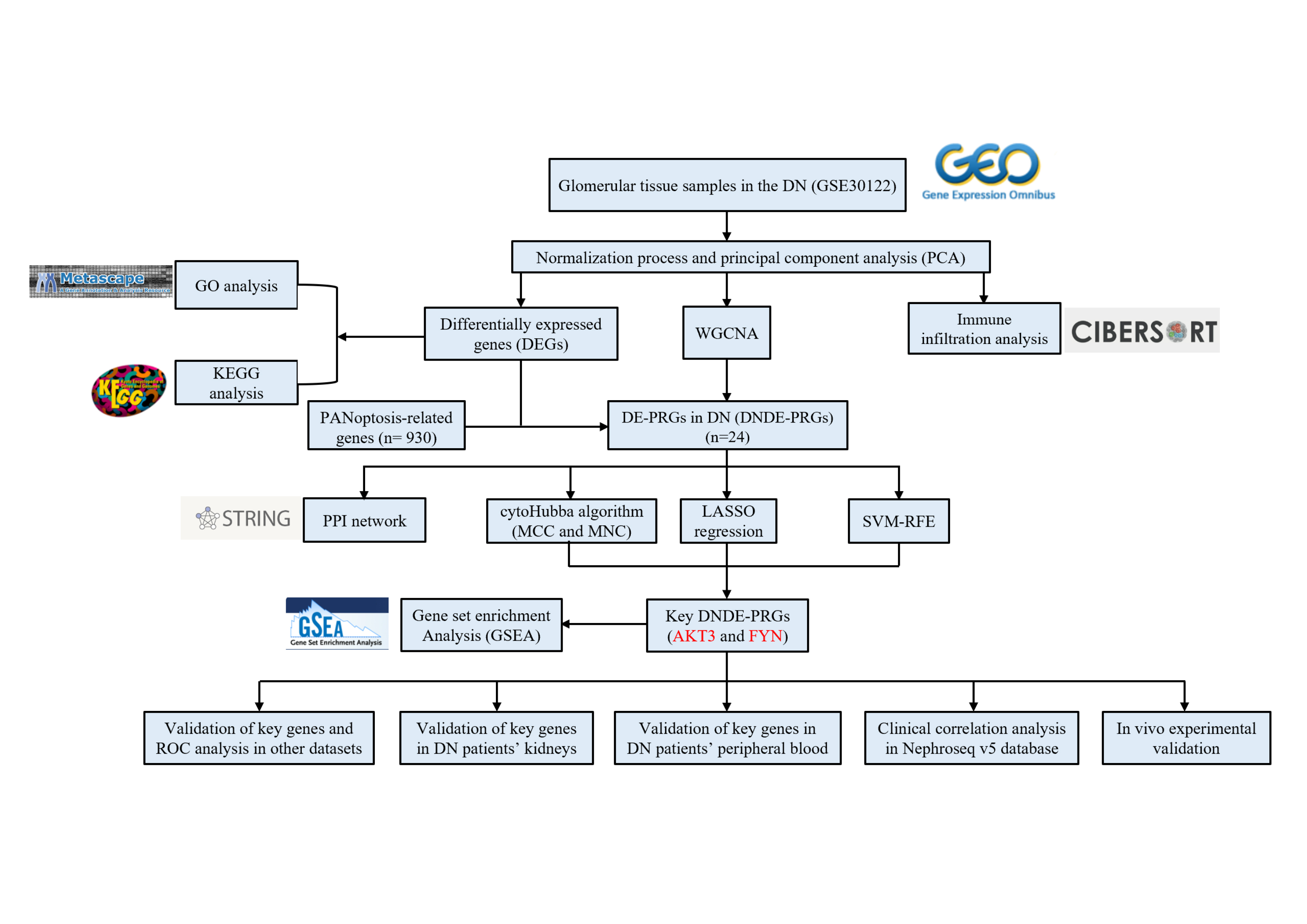

Supplement: Supplementary file 1 [file DataSheet1.zip › Supplementary files/Fig S1.jpg]

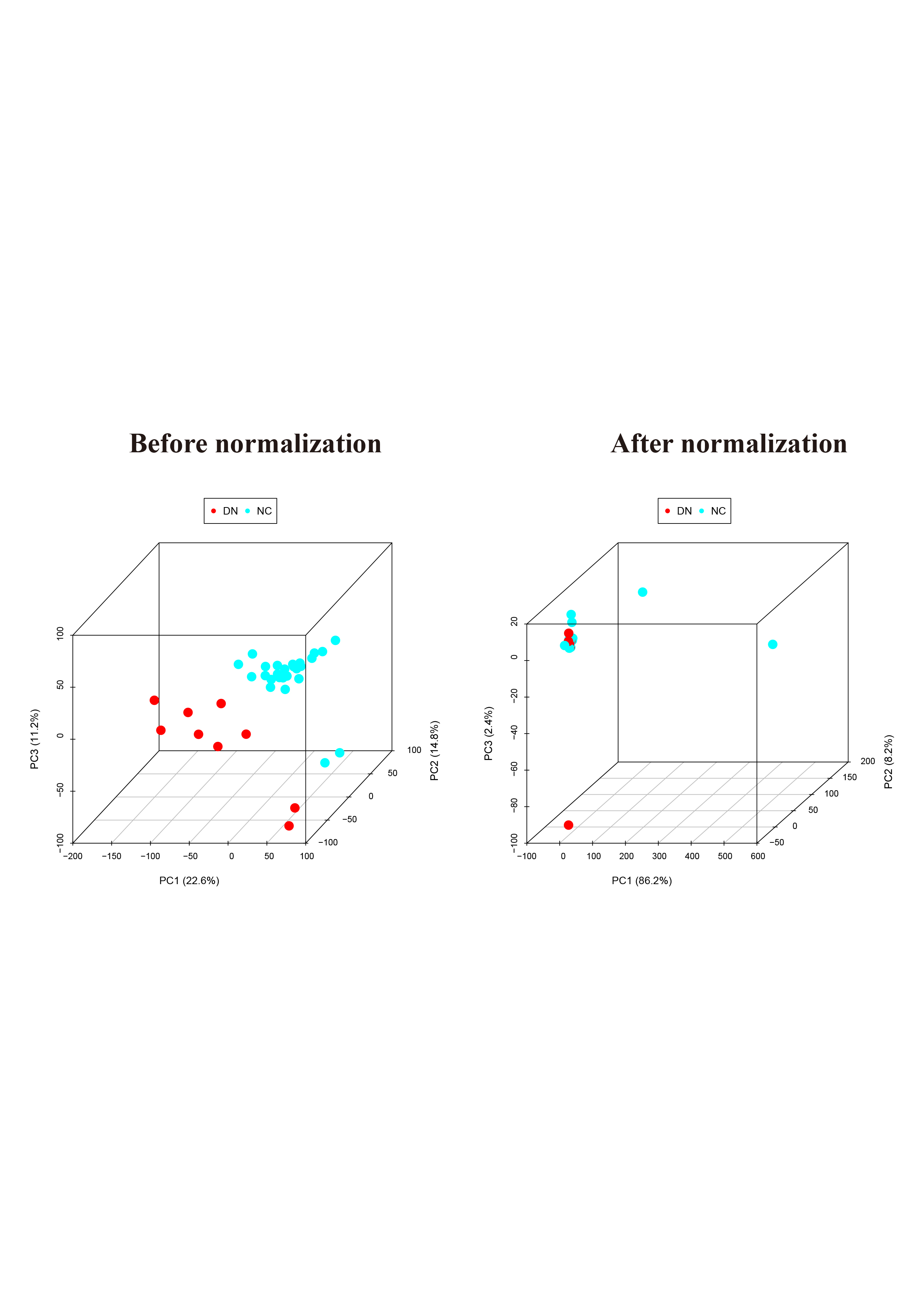

Supplement: Supplementary file 1 [file DataSheet1.zip › Supplementary files/Fig S2.jpg]

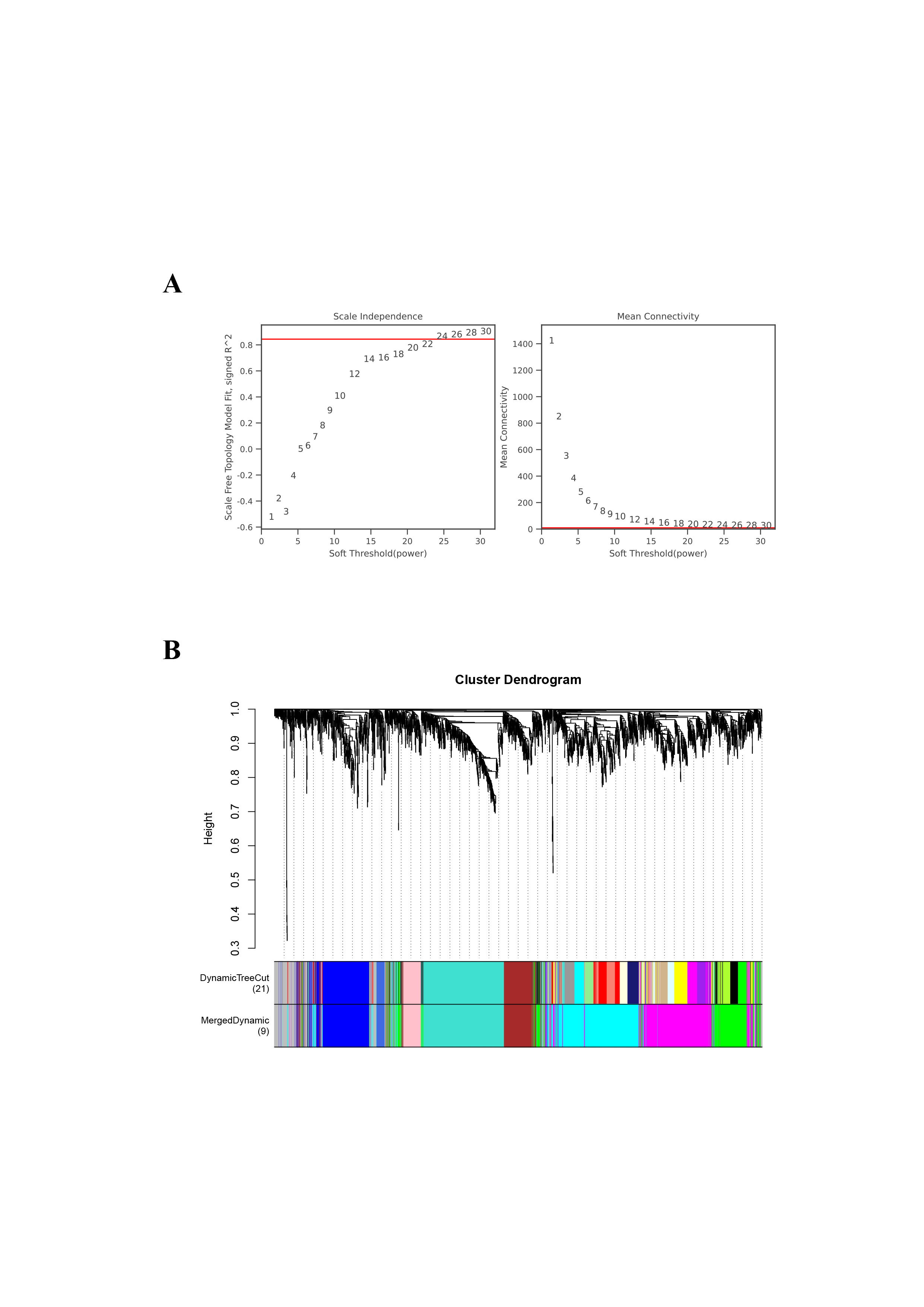

Supplement: Supplementary file 1 [file DataSheet1.zip › Supplementary files/Fig S3.jpg]

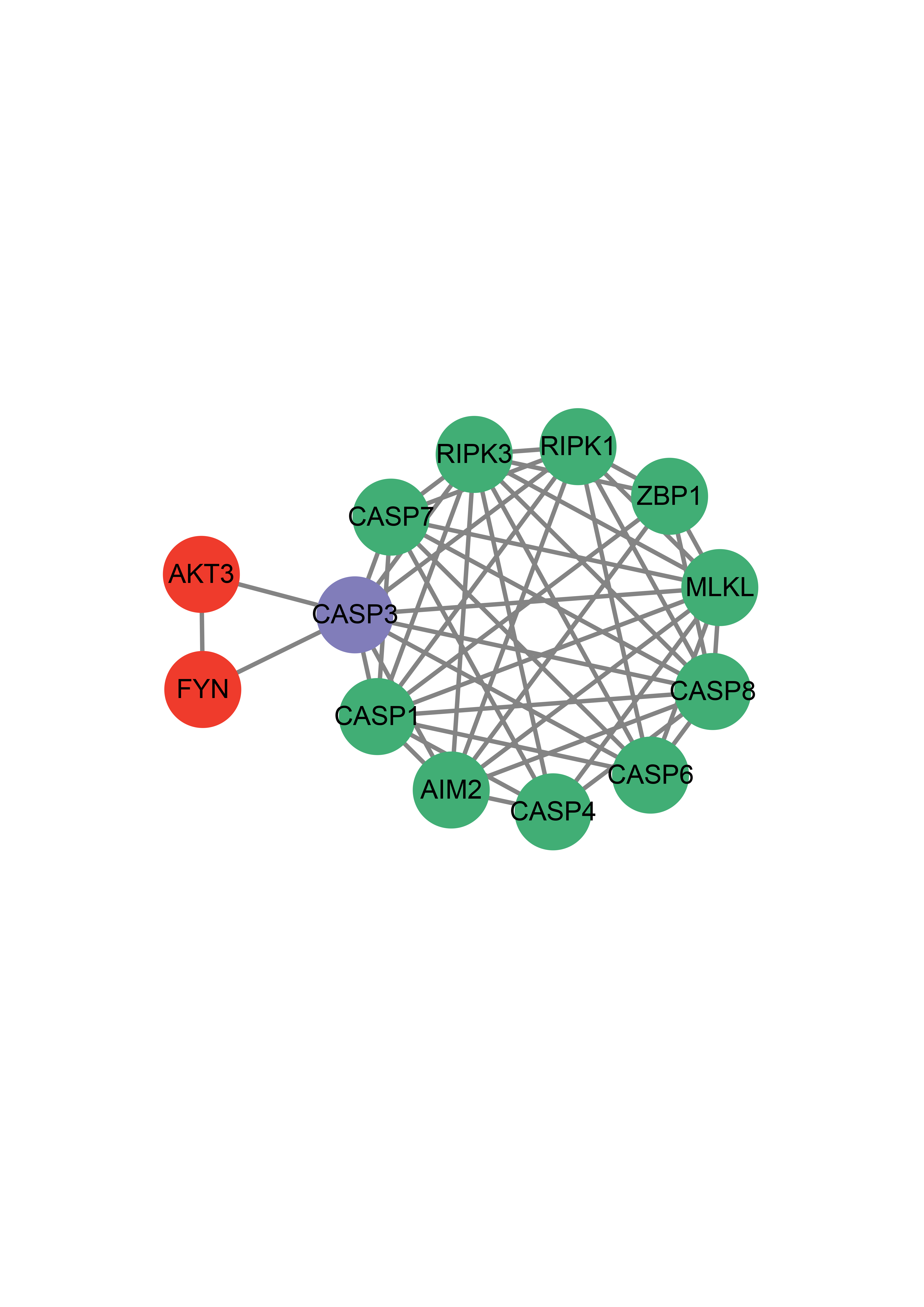

Supplement: Supplementary file 1 [file DataSheet1.zip › Supplementary files/Fig S4.jpg]
